# 胸腔镜肺癌肺切除术后患者住院时间延长（ > 7天）的病因分析——附115例报道

**DOI:** 10.3779/j.issn.1009-3419.2018.03.23

**Published:** 2018-03-20

**Authors:** 亮 戴, 晓征 康, 万璞 闫, 永波 杨, 培俍 赵, 浩 付, 海涛 周, 震 梁, 宏超 熊, 瑶 林, 克能 陈

**Affiliations:** 100142 北京，北京大学肿瘤医院暨恶性肿瘤发病机制及转化研究教育部重点实验室，胸外一科 Key Laboratory of Carcinogenesis and Translational Research (Ministry of Education), the First Department of Thoracic Surgery, Peking University Cancer Hospital and Institute, Peking University School of Oncology, Beijing 100142, China

**Keywords:** 肺肿瘤, 电视胸腔镜手术, 并发症, 住院时间, Lung neoplasms, Video assisted thoracic surgery, Complications, Hospitalization

## Abstract

**背景与目的:**

胸腔镜手术已是我科肺癌肺切除手术的主要方式，其特点是创伤小、恢复快，术后7天以内出院患者近90%，但术后并发症仍时有发生；我们对胸腔镜肺癌肺切除术后住院时间 > 7天的患者进行分析，旨在总结并发症的种类及危险因素，提高患者的术后安全性。

**方法:**

数据来源为北京肿瘤医院胸外一科前瞻性肺癌数据库，选取2010年1月-2014年12月行胸腔镜肺癌肺切除手术，且住院时间 > 7天的患者，调查其并发症种类，并按照改良Clavien分级将其分为轻度及重度并发症，分析临床因素与并发症程度之间的关系。

**结果:**

术后住院时间 > 7天者共115例，占同期手术比例为10.3%（115/1, 112）。全组患者轻度并发症81例，占同期手术（1, 112例）比例和术后住院时间延长者比例分别为7.3%和70.4%，重度并发症34例，分别为3.1%和29.6%；因并发症死亡者2例，分别为0.18%和1.7%；术后最常见者为漏气超过5日20例，分别为1.8%和17.4%，肺不张19例，分别为1.7%和16.5%，肺部感染18例，分别为1.6%和15.7%；罕见并发症中支气管胸膜瘘4例（0.36%和3.5%），但危险高，其中2例因并发急性呼吸窘迫综合征（acute respiratory distresssyndrome, ARDS）围手术期死亡；临床因素中仅低肺功能（FEV_1_% < 70%）可能是造成术后严重并发症的因素（45.8% *vs* 23.6%, *P*=0.038）；轻度并发症组与重度并发症组5年无疾病生存率（52.2% *vs* 51.9%, *P*=0.894）及5年总生存率（64.0% *vs* 53.5%, *P*=0.673）均无显著差异。

**结论:**

术后持续漏气、肺不张及肺部感染等并发症是延长胸腔镜肺癌术后住院时间的主要原因，而支气管胸膜瘘是最凶险的并发症；术前低肺功能患者更易出现术后严重并发症，但并发症严重程度并不会影响远期预后。

## Abstract (en)

**Background and objective:**

Thoracoscopic surgery has gradually become the major procedure for lung cancer surgery in our department. Its characteristics are minimal trauma and quick recovery, which make approximately 90% of patients discharge from the hospital after surgery. However, the postoperative complications still happen now and then. We analyzed the patients who had been hospitalized for longer than 7 days after thoracoscopic lung cancer surgery, aiming to summarize the types and risk factors of complications, and improve postoperative safety of patients.

**Methods:**

The data were come from the prospective database of Thoracic Surgery Unit One in Peking Cancer Hospital, and patients that underwent thoracoscopic pulmonary surgery between Jan. 2010 and Dec. 2014 with length of stay more than 7 days were included in the study. The classifications of the complications were investigated and graded as mild or severe complications according to modified Claviengrading, the relationship between clinical factors and degrees of complications was also analyzed.

**Results:**

The hospitalization of 115 cases were longer than 7 days after surgery, accounting for 10.3% (115/1, 112) of the whole patients that underwent surgery during the same period. Eighty-one cases had mild complications, accounting for 7.3% (81/1, 112) of the whole cases that underwent surgery during the same period and 70.4% (81/115) of the cases with prolonged length of stay; the proportions of severe complications in both groups were 3.1% (34/1, 112) and 29.6% (34/115), respectively; and the proportions of complications that caused perioperative deaths were 0.18% (2/1112) and 1.7% (2/115), respectively. Among all the postoperative complications, the most common was air leakage for more than 5 days after surgery, with a total of 20 cases (1.8% and 17.4%). The other common complications were: atelectasis (19 cases, 1.7% and 16.5%), pulmonary infection (18 cases, 1.6% and 15.7%), etc. The less common complications was bronchopleural fistula (4 cases, 0.36% and 3.5%) with very high risk, and 2 cases died perioperatively due to the combination of acute respiratory distresssyndrome (ARDS). In the clinical factors, only preoperative low pulmonary function (FEV_1_% < 70%) was the potential risk factor for postoperative severe complications (45.8% *vs* 23.6%, *P*=0.038). There was no significant difference either regarding the 5 year disease free survival or the 5 year overall survival between mild complication group and severe complication group, with 5 year DFS being 52.2% and 51.9%, respectively (*P*=0.894), and 5 year overall survival being 64.0% and 53.5%, respectively (*P*=0.673).

**Conclusions:**

Continuous postoperative air leakage, atelectasis and pulmonary infections were the major causes for prolonged hospitalization after thoracoscopic surgery for lung cancer, and bronchopleural fistula was the most perilous complications. Patients with low preoperative pulmonary function were more likely to have severe postoperative complication, however, this would not influence the long term survival of the patients.

肺癌已经成为世界范围内最常见和致死率最高的恶性肿瘤^[1]^。尽管非小细胞肺癌强调根据分期不同采用多学科、多程综合治疗，然而，外科手术仍在非小细胞肺癌治疗中有着重要的地位。肺癌外科的手术方式随着医学科技的发展，手术器械的革新，在近20年有了长足的进步^[2]^。胸腔镜手术以其创伤小、恢复快、操作简便以及视野清晰等优势，逐渐取代了传统的开胸手术^[3, 4]^。我科自2002年逐步开展肺癌胸腔镜手术后，术后平均住院日由传统开胸手术的7天-10天，缩短到现在的3.5天，但仍有部分住院时间延长者，这往往是由于术后并发症导致。分析术后住院时间延长患者并发症的种类及危险因素，能够帮助预防及干预此类患者相关并发症，加速术后康复^[5]^。本文就本中心单一医生组同期1, 112例肺癌胸腔镜肺切除手术患者中115例因并发症术后住院时间超过7天者的并发症严重程度、种类、发生率以及危险因素进行回顾性分析，以供大家参考。

## 1 对象与方法

### 1.1 研究对象

我科肺癌前瞻性数据库始建于2000年，胸腔镜用于肺癌外科治疗开始于2002年，至2010年后各种全腔镜肺癌术式已成熟，故本研究选取数据库中2010年1月至2014年12月连续的行根治性手术的肺癌患者1, 112例。入组标准：①肺原发恶性肿瘤者；②行胸腔镜根治性手术治疗者；③术后住院时间因并发症超过7天者。排除标准：①原发灶病理类型为小细胞肺癌者；②既往恶性肿瘤病史者；③术前美国东部肿瘤协作组（Eastern Cooperative Oncology Group, ECOG）评分≥2者；④腔镜术中中转开胸者。

### 1.2 诊断与治疗策略

肿瘤分期检查包括胸部增强计算机断层扫描（computed tomography, CT）、全身全身正电子发射计算机断层显像（positron emission tomography/CT, PET/CT）、头颅增强磁共振成像（magnetic resonance imaging, MRI）、全身骨扫描及气管镜检查，对于怀疑纵隔淋巴结阳性者行支气管内超声（endobroncheal ultrasonography, EBUS）穿刺或电视纵隔镜检查。肿瘤分期采用2009年国际抗癌联盟（Union for International Cancer Control, UICC）和美国癌症联合会（American Joint Committee on Cancer, AJCC）联合制定的第7版TNM分期系统^[6]^。部分肿瘤长径≥4 cm或cN+者术前给予含铂两药方案新辅助化疗。手术均为全腔镜下操作，以根治性为目的，术式包括亚肺叶切除术、肺叶切除术、支气管袖状切除术及全肺切除术，部分亚肺叶切除者行淋巴结采样，其余均行系统性淋巴结清扫术。

### 1.3 围手术期并发症的定义、评估及分级

围手术期并发症的定义和分类标准采用美国胸外科医师协会（Society of Thoracic Surgeons, STS）普胸外科数据库（General Thoracic Surgery Database, GTSD）的定义^[7]^。并发症分级采用2004年制定的改良Clavien并发症分级系统^[8]^。Clavien 1级-2级并发症定义为轻度并发症，Clavien 3级-5级并发症定义为严重并发症。由于本前瞻性数据库中无并发症严重程度的分级，故此次分级由科室多名高年资医师相互独立分级，对有争议者引入第三组医师，疑难者行全科讨论。

### 1.4 随访方式

术后2年之内每3个月复查一次，第3年至第5年每半年复查一次，5年以后每年复查一次。术后检查内容包括胸部增强CT、头颅增强MRI/CT、支气管镜、全身骨扫描、锁骨上区及腹部超声及全身PET/CT。部分患者根据症状及检查结果行经支气管针吸活检（transbronchial needle aspiration, TBNA）、EBUS穿刺或纵隔镜。本组患者随访截止日期为2018年1月15日或死亡，随访率100%；中位随访时间18.1个月（6.2个月-149.8个月）。

### 1.5 研究指标

主要包括患者一般信息（性别、年龄、吸烟史），术前肺功能、术前合并症，手术信息，肿瘤病理及分期，术后并发症（种类、分级），术后住院时间，无病生存时间及总生存时间等。

### 1.6 统计学方法

统计分析采用SPSS 22.0分析（SPSS公司，芝加哥，伊利诺斯，美国）。临床因素组间并发症程度比较采用卡方检验；单因素生存分析采用*Kaplan-Meier*法，用*Log-rank*进行显著性检验。统计结果均按照*P* < 0.05定义为差异具有统计学意义。

## 2 结果

### 2.1 基本资料

本研究最终纳入115例，中位年龄为65岁，27岁-82岁，男性81例（70.4%），女性34例（29.6%）；吸烟患者76例（66.1%），非吸烟患者39例（33.9%）；术前行新辅助化疗者25例（21.7%），直接手术者90例（78.3%）；手术行亚肺叶切除者10例（8.7%），单纯肺叶切除者87例（75.7%），复杂肺叶切除者16例（13.9%），全肺切除者2例（1.7%）；术后病理为腺癌者74例（64.3%），鳞癌者30例（26.1%），其他类型者11例（9.6%）；根据AJCC/UICC第7版TNM分期系统，术后病理分期为Ⅰ期者81例（70.4%），Ⅱ期者16例（13.9%），Ⅲ期期者15例（13.0%），Ⅳ期者3例（2.6%）；全组中位术后住院时间为10天（8天-61天）（表 1）。

**1:** 患者基本资料 Baseline characteristics of patients

	*n*	Complications		*P*
		Clavien 1-2	Clavien 3-5	
No. of patients	115	81 (70.4%)	34 (29.6%)	
Age (yr)				0.723
＜70	75	52 (69.3%)	23 (30.7%)	
≥70	40	29 (72.5%)	11 (27.5%)	
Gender				
Male	81	57 (70.4%)	24 (29.6%)	0.981
Female	34	24 (70.6%)	10 (29.4%)	
Smoking				0.509
Yes	76	52 (68.4%)	24 (31.6%)	
No	39	29 (74.4%)	10 (25.6%)	
FEV_1_%				0.038
＜70%	24	13 (54.2%)	11 (45.8%)	
≥70%	72	55 (76.4%)	17 (23.6%)	
Comorbidity				
HTN	42	27 (64.3%)	15 (35.7%)	0.273
COPD	3	1 (33.3%)	2 (66.7%)	0.154
CAD	8	5 (62.5%)	3 (37.5%)	0.610
DM	10	8 (80.0%)	2 (20.0%)	0.488
Histology				0.470
Ad	74	55 (74.3%)	19 (25.7%)	
Sq	30	19 (63.3%)	11 (36.7%)	
Others	11	7 (70.4%)	4 (29.6%)	
pTNM stage				0.191
Stage Ⅰ	81	53 (65.4%)	28 (34.6%)	
Stage Ⅱ	16	13 (81.3%)	3 (18.7%)	
Stage Ⅲ/Ⅳ	18	15 (83.3%)	3 (16.7%)	
HTN: hypertention; COPD: chronic obstructive pulmonary disease; CAD: coronary artery disease; DM: diabetes mellitus.				

### 2.2 115例术后住院时间 > 7天患者并发症分析

全组患者术后住院时间 > 7天者共115例，占同期手术者比例为10.3%（115/1, 112）。根据改良Clavien并发症分级系统，轻度并发症（Clavien 1级-2级）占同期手术比例和占术后住院时间延长者比例分别为7.3%（81/1, 112）和70.4%（81/115），重度并发症占比分别为3.1%（34/1, 112）和29.6%（34/115），其中并发症致围手术期死亡者占比分别为0.18%（2/1, 112）和1.7%（2/115）。所有术后并发症中最常见为术后漏气超过5日，共20例，其他常见并发症为肺不张19例，肺部感染18例，心房颤动17例，胸腔积液14例，心肌梗死7例，皮下气肿6例；不常见并发症有肺栓塞1例，乳糜胸2例，支气管胸膜瘘4例，其中2例因并发急性呼吸窘迫综合征（acute respiratory distresssyndrome, ARDS）出现呼吸衰竭围手术期死亡（表 2）。

**2:** 患者术后并发症发生情况 Details of postoperative complications

	*n*	Rate
Persistent air leak ( > 5 d)	20	1.8%
Atelectasis	19	1.7%
Pneumonia	18	1.6%
Atrial fibrillation	17	1.5%
Pleural effusion(≥Middle-plenty)	14	1.3%
Myocardial infarction	7	6.3‰
Emphysema	6	5.4‰
Bronchopleural fistula	4	3.6‰
Chylothrax	2	1.8‰
Pulmonary embolism	1	0.9‰

### 2.3 严重并发症患者危险因素分析

我们将可能引起术后严重并发症的因素分为一般因素和治疗相关因素，一般因素包括年龄、性别、是否吸烟、肺功能、合并症（高血压，慢性阻塞性肺病，冠状动脉疾病和糖尿病）、病理类型及病理分期，其中仅肺功能与并发症严重程度相关，FEV_1_% < 70%者出现术后严重并发症概率更高（45.8% *vs* 23.6%, *P*=0.038），而既往COPD患者似乎更易发生严重并发症（66.7% *vs* 33.3%），但*P*=0.154；治疗相关因素包括是否新辅助化疗、手术术式、手术时间、麻醉评分及是否术中输血，结果手术术式中单纯肺叶切除比亚肺叶切除及复杂肺叶切除更容易出现严重并发症（36.8% *vs* 10% *vs* 5.65, *P*=0.011），麻醉评分2级/3级者（34.1% *vs* 16.7%）及术中输血者（60.0% *vs* 28.25%）出现术后严重并发症概率似乎更高，但差异均无统计学意义（表 3）。

**3:** 不同程度术后并发症间治疗差异比较 Comparison of the therapy between different degrees of postoperative complications

	*n*	Complications		*P*
		Clavien 1-2	Clavien 3-5	
Neoadjuvant chemotherapy				0.846
Yes	25	18 (72.0%)	7 (18.0%)	
No	90	63 (70.0%)	27 (30.0%)	
Surgical procedure				0.011
Sublobectomy	10	9 (90.0%)	1 (10.0%)	
Lobectomy	87	55 (63.2%)	32 (36.8%)	
Bronchial sleeve resection/pneumonectomy	18	17 (94.4%)	1 (5.6%)	
Operation time				0.244
< 135 min	57	43 (75.4%)	14 (24.6%)	
≥135 min	58	38 (65.5%)	20 (34.5%)	
ASA				0.072
Status 1	30	25 (83.3%)	5 (16.7%)	
Status 2/3	85	56 (65.9%)	29 (34.1%)	
Intraoperative blood transfusion				0.127
Yes	5	2 (40.0%)	3 (60.0%)	
No	110	79 (71.8%)	31 (28.2%)	
ASA: American Society of Anesthesiologists.				

### 2.4 影响115例术后住院延长患者远期预后的因素

本组患者中位无疾病生存时间为68.4个月（95%CI：43.1个月-93.8个月），5年总生存率为62.3%。按照术后并发症严重程度进行分层分析，轻度并发症者与重度并发症者5年无疾病生存率分别为52.2% *vs* 51.9%，*P*=0.894；两者5年总生存率分别为64.0% *vs* 53.5%，*P*=0.673，术后并发症严重程度并不会影响远期预后（图 1）。

**1:**
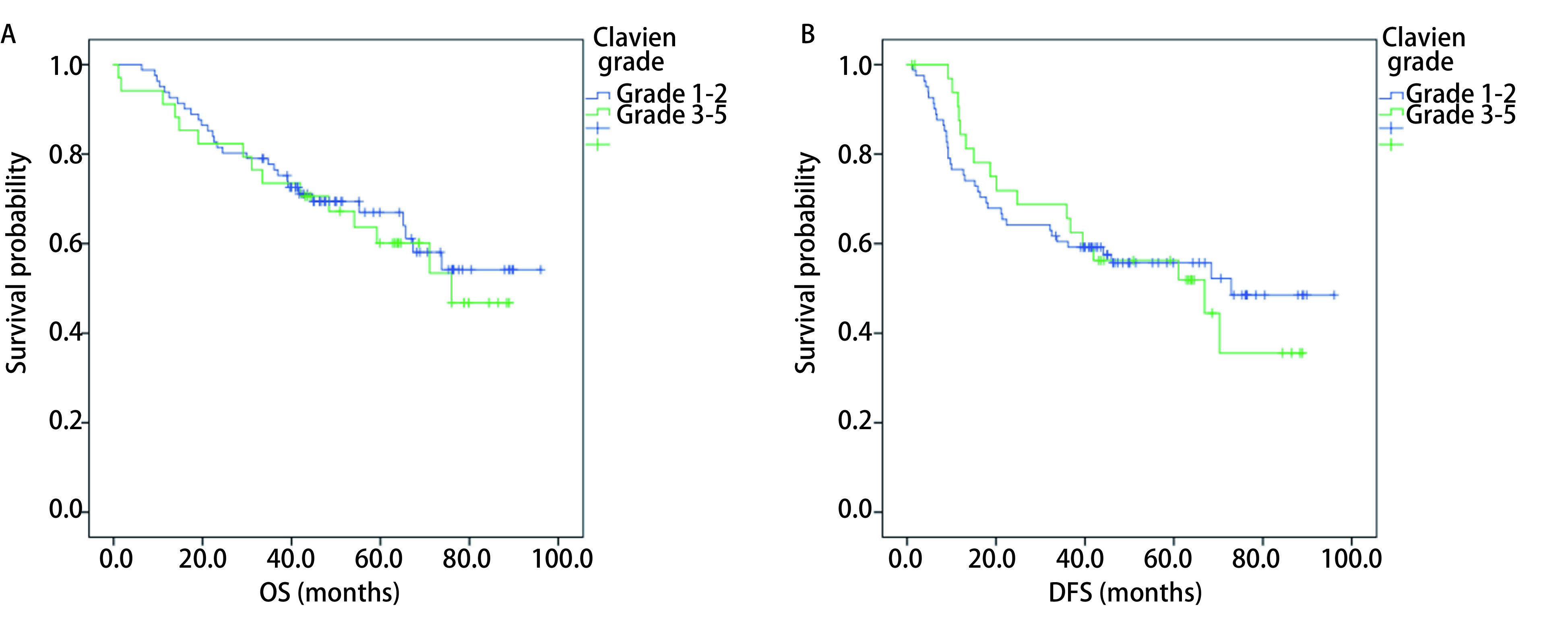
不同并发症严重程度分级患者OS（A）及DFS（B）*Kaplan-Meier*曲线 OS (A) and DFS (B) *Kaplan-Meier* curves in patients with different grade of postoperative complications. OS: overall survival; DFS: disease-free survival.

## 3 讨论

胸腔镜肺手术开始于20世纪90年代初期，历经近30年发展，其适应证由最初良性疾病的楔形切除，发展到如今包括支气管、血管袖式切除在内的各式复杂肺癌手术，已成为肺癌外科首选的术式^[9]^。随着胸腔镜肺手术技术的不断进步，在等同常规开胸手术肿瘤学治疗效果的前提下，能够使手术时间更短、创伤更小，让患者痛苦更轻、并发症更少且恢复更快^[10]^。值得一提的是，随着多学科的进步、手术技术的提高、围手术期处理保证，以年龄、器官功能、合并疾病为组合考虑的手术适应症在不断扩大，手术禁忌证正在不断缩小，高风险患者接受手术的机会越来越多。这一方面为那些肿瘤学边缘（分期较晚）或外科学边缘（高龄或器官功能异常）的患者提供了手术机会，但另一方面也是造成手术死亡增高、并发症增加的直接原因^[11]^。如何更好的减少术后并发症，缩短术后住院时间，是所有胸外科医师不断追求的目标。因此，我们回顾性分析胸腔镜肺癌术后因并发症导致住院时间延长的患者，希望从失败中找寻原因，通过加强围手术期管理，进一步提高胸腔镜肺癌手术质量，降低术后严重并发症。

1992年Clavien等^[12]^提出评估并发症严重程度的标准-Clavien并发症分级系统（简称Clavien分级）。该系统将手术相关不良事件分为并发症（complication）、治疗失败（failure to cure）及后遗症（sequela）三类，并按并发症严重程度及所需处理措施将并发症分为四个级别。2004年Dindo等^[8, 13]^在总结6, 336例患者手术并发症资料的基础上对此系统进行了更新，制定了改良Clavien并发症分级系统，并经后续研究证明改良系统的客观性及可重复性较突出，便于不同医院之间进行手术疗效分析以及并发症的对比。我们分析了肺癌术后因并发症住院时间延长的115例患者，通过比较其一般资料及治疗相关信息，期望找到术后严重并发症（改良Clavien分级3级-5级）的有关因素。结果显示在众多可能的因素中低肺功能（FEV_1_% < 70%）可能是造成术后严重并发症的因素；而其他常见的因素，如高龄、吸烟、术前合并症、新辅助化疗及手术时间长等均不是严重并发症的相关因素。原因可能是充足的术前准备，术中严格操作及围手术期管理的加强，避免了这些不良因素对术后并发症的影响，使得整组患者严重并发症发生率仅3.1%。

尽管如此，但为了更好的降低肺癌术后并发症发生率，几种本研究常见的和危害较大的并发症也值得我们进一步的探讨。持续漏气指是肺切除术后最常见的并发症之一。几乎所有接受肺叶切除的病人都有一定程度的术后漏气，临床表现为胸腔引流系统持续气泡，但大部分漏气源于肺实质裁制切缘或分离叶间裂时肺破损处，多在手术后24 h-48 h内剩余肺组织复张到完全填满胸腔^[14]^。如果漏气持续超过5天-7天，则认为是持续的，会增加并发症率和延长住院时间^[15]^。持续漏气如若引流不畅，还会引起皮下、纵隔气肿，甚至张力性气胸危及生命。本组患者术后最常见并发症为持续漏气超过5天，共20例，均为分离叶间裂处肺破损引起，虽然并未引起严重并发症，但增加了患者引流管带管时间，延长了住院时间，手术中对于叶间裂发育欠佳者，叶间裂处理可采用腔镜下切割闭合器，对于试水漏气处应缝合。

另外一项引起住院时间延长的常见并发症是肺不张，肺切除术后肺不张通常见于术前有肺部疾病合并症、肺功能较差者，由于术中及术后呼吸道分泌物清理不足（术中吸痰不佳、术后拔管前未肺复张、术后咳嗽差、不活动等），导致残余肺实质通气不足而引起的，加强术前呼吸道准备，手术麻醉结束前呼吸机肺复张，以及术后早期下床活动可以减少肺不张的发生。本组19例肺不张患者中有10例为右肺术后出现的右肺中叶不张，经过多次床旁支气管镜吸痰后均复张良好。另外，支气管袖状切除术后由于吻合部位上皮细胞的血管及淋巴管破坏造成局部水肿，或吻合肺叶部分去神经化造成排痰不畅，会有5%-10%的患者出现术后肺不张^[16]^。本研究出现的1例右肺上叶袖式切除后右肺中叶不张，经吸痰等保守治疗2周后逐渐复张。

支气管胸膜瘘是一项紧急的、严重的肺切除术后并发症，可在术后任何时间发生，但最常发生在术后8天-12天内。突发的支气管胸膜瘘会因胸腔积液突然大量进入呼吸道造成误吸，或因张力性气胸而危及生命^[17]^。全肺切除术后支气管胸膜瘘发生率高于肺叶切除术和袖式切除术，但后两者支气管胸膜瘘发生率相当。所有形式的肺切除术后，支气管胸膜瘘发生率在2%-13%之间，死亡率为30%-70%^[18-20]^。本组患者中，虽然支气管胸膜瘘发生率仅为0.36%，但发生后死亡率高达50%，均为误吸造成肺部感染导致呼吸衰竭死亡。特别是肺癌术后住院时间不断缩短，晚期瘘的患者出院后很难发现，且容易因抢救不及时耽误治疗。因此，围手术期保证充足营养，术中尽量缩短支气管残端，保证残端切缘净，术中严格试水漏气试验，并且对术后胸片持续出现液气胸，或伴有持续白细胞升高者高度警惕，积极行支气管镜检查，能够有效避免及早期发现支气管胸膜瘘。

术后并发症的发生原因包括患者本身重要脏器的功能、疾病本身的性质与范围（TNM分期）、以及治疗措施的得当与否。选择合适生理条件的病人，较早期的病变，以及掌握良好的外科技术是降低手术死亡的重要前提。严于术前准备，精于术中操作，善于术后管理是降低食管癌手术并发症和死亡的保证。同时，客观、准确、适时的分析既往并发症的原因及相关因素有助于我们更好的避免其发生，增加患者治疗安全性。

## References

[1] Siegel R, Ma J, Zou Z (2014). Cancer statistics, 2014. CA Cancer J Clin.

[2] Jr MKR, Houck W, Fuller CB (2006). Video-assisted thoracic surgery lobectomy: experience with 1, 100 cases. Ann Thorac Surg.

[3] Paul S, Altorki NK, Sheng S (2010). Thoracoscopic lobectomy is associated with lower morbidity than open lobectomy: a propensity-matched analysis from the STS database. J Thorac Cardiovasc Surg.

[4] Falcoz PE, Puyraveau M, Thomas PA (2016). Video-assisted thoracoscopic surgery versus open lobectomy for primary non-small-cell lung cancer: a propensity-matched analysis of outcome from the European Society of Thoracic Surgeon database. Eur J Cardiothorac Surg.

[5] Kozower BD, Sheng S, O'Brien SM (2010). STS database risk models: predictors of mortality and major morbidity for lung cancer resection. Ann Thoracic Surg.

[6] Edge SB, Compton CC (2010). The American Joint Committee on Cancer: the 7th Edition of the AJCC Cancer Staging Manual and the Future of TNM. Ann Surg Oncol.

[b7] 7Society of Thoracic Surgeons 2010. Available at: http://www.sts.org/sections/stsnationaldatabase/. Accessed January 4, 2010.

[8] Dindo D, Demartines N, Clavien PA (2004). Classification of surgical complications: a new proposal with evaluation in a cohort of 6336 patients and results of a survey. Ann Surg.

[9] Shaheen S, Jabo B, Kaur M (2017). P1.16-006 Less Is More: video assisted thoracic surgery (VATS) *vs* open thoracotomy in the management of resectable lung cancer. J Thorac Oncol.

[10] Yang CJ, Kumar A, Klapper JA (2017). A national analysis of long-term survival following thoracoscopic versus open lobectomy for stage Ⅰ non-small-cell lung cancer. Ann Surg.

[11] Wright C D, Gaissert H A, Grab J D (2008). Predictors of prolonged length of stay after lobectomy for lung cancer: a Society of Thoracic Surgeons General Thoracic Surgery Database risk-adjustment model. Ann Thorac Surg.

[12] Clavien PA, Sanabria JR, Strasberg SM (1992). Proposed classification of complications of surgery with examples of utility in cholecystectomy. Surgery.

[13] Clavien PA, Barkun JMD, De Oliveira ML (2009). The Clavien-Dindo Classification of surgical complications: five-year experience. Ann Surg.

[14] Kim EA, Lee KS, Shim YM (2002). Radiographic and CT findings in complications following pulmonary resection. Radiographics.

[15] Venuta F, Rendina EA, De Giacomo T (2010). Postoperative strategies to treat permanent air leaks. Thorac Surg Clin.

[16] Massard G, Wihlm JM (1998). Postoperative atelectasis. Chest Surg Clin N Am.

[17] Lois M, Noppen M (2005). Bronchopleural fistulas: an overview of the problem with special focus on endoscopic management. Chest.

[18] Tabutin M, Couraud S, Guibert B (2012). Completion pneumonectomy in patients with cancer: postoperative survival and mortality factors. J Thorac Oncol.

[19] Hollaus PH, Lax F, El-Nashef BB (1997). Natural history of bronchopleural fistula after pneumonectomy: a review of 96 cases. Annals of Thoracic Surgery.

[20] Sirbu H, Busch T, Aleksic I (2001). Bronchopleural fistula in the surgery of non-small cell lung cancer: incidence, risk factors, and management. Ann Thorac Cardiovasc Surg.

